# Transurethral 160-W straight beam green laser vaporesection of the prostate: initial experience after 180 procedures

**DOI:** 10.1186/s40064-016-1776-6

**Published:** 2016-07-04

**Authors:** Lianjun Li, Bo Hu, Muwen Wang, Peng Sun, Xunbo Jin

**Affiliations:** Minimally Invasive Urology Center, Shandong Provincial Hospital Affiliated to Shandong University, 324# Jingwu Road, Jinan, 250021 Shandong Province China

**Keywords:** Prostate, Benign prostatic hyperplasia, LBO laser, Photoselective vaporization, Laser prostatectomy, Photoselective vaporesection

## Abstract

Although the photoselective vaporization of the prostate has been considered one of the most promising alternatives for treatment of benign prostatic hyperplasia (BPH), published clinical data with the surgical technology of straight beam lithium triborate laser (LBO) is still lacking. To evaluate the technical improvement and initial experience of the 160-W straight beam LBO laser photoselective vaporesection of the prostate (PVRP) for the surgical treatment of BPH. From September 2012 to September 2014, including a 12-month follow-up, a prospective randomized study was performed. 180 patients undergoing PVRP were included in the study. All patients were preoperatively assessed by International Prostate Symptom Score (IPSS), maximum flow rate (Qmax), post-void residual urine (PVR), prostate-specific antigen level, and prostate volume measurement. Perioperative parameters and complications were recorded. Patients were reassessed at 1, 3, 6 and 12 months postoperatively. PVRP resulted in a significant improvement of IPSS, Qmax, and PVR. Mean operative time was 48.3 ± 14.4 min. A significant improvement for PVRP was achieved regarding the catheter indwelling and hospital stay time. No severe perioperative complications were recorded. No requiring blood transfusion in all patients. Capsule perforation was observed in four patients in the group. There were four patients experienced bladder neck contracture and another four patients were diagnosed urethral stricture, all of whom were treated well by dilatation finally without reoperation. 160-W straight beam LBO laser PVRP appears to be a feasible and safe alternative for symptomatic BPH with decreased length of catheter indwelling and hospital stay time postoperatively.

## Background

Reza Malek first reported photoselective vaporization of the prostate (PVP) of benign prostatic hyperplasia (BPH) using the potassium-titanyl-phosphate (KTP) 80-W system in 1996 (Malek et al. 2005). Since then the surgical technique has been a rapid development. The 120-W green laser device used to treat BPH was first reported in 2006 (Hermanns et al. 2011; Ben-Zvi et al. 2013). The application report of 180-W XPS system indicated that the power increased substantially (Bachmann et al. 2012, 2014). Furthermore, Yong-Guang Gong reported the 120-W front-fire lithium triborate laser (LBO) photoselective vaporesection of the prostate (PVRP) for the surgical treatment of BPH (Gong et al. 2014). These improvement greatly enhance the operation efficiency and therefore reduce the risk of surgery. Increasing study over the past 10 years indicates that PVP with green laser is equally effective as trans-urethral resection of prostate (TURP) in BPH treatment (Chen et al. 2012; Emara and Barber 2014; Teng et al. 2013). Above all, the green laser vaporization surgery can strongly challenge the status of TURP as the gold standard in surgical treatment of BPH.

There have been reported that the technologies of PVP has had specific benefits over TURP in terms of shorter duration for catheterization and hospital stay (Guo et al. 2015; Chen et al. 2012). Despite these advantages, the shortcomings of conventional PVP are obvious, even for experienced surgeons of green laser operations. Compared with TURP, the main problems of traditional PVP surgery include that wash water circulation is poor which cause unclear visualization, speed of vaporization is too slow, the apex of prostate is more likely to have residual gland, and bleeding during the surgery is difficult to be stopped, some of which problems have been improved by using of 180 W XPS system (Schwartz et al. 2011; West and Woo 2015).

Based on these reasons, we use trans-urethral plasma kinetic resection instrument to perform green laser surgery in order to improve the rinse water to achieve a clear vision. Furthermore, we proposed the method of photoselective retrograde stripping-vaporization of the prostate (PRSVP), which is a new form of PVRP. Currently, there is still little experience reported on the new straight beam LBO laser. We summarize the data from 180 patients treated from 2012 to 2014, including a 12-month follow-up, so that to evaluate the safety and efficacy of PRSVP using the 160-W straight beam LBO laser.

## Methods

This prospective study was performed between September 2012 and September 2014 in Provincial Hospital Affiliated to Shandong University. A total of 180 patients suffering from BPO due to BPH and failed previous medical therapy were included in this study. The patients were treated with the 160 W straight beam LBO laser PRSVP by one experienced urologist in our department. Written consent was obtained from all participants and the protocol was approved by the ethics committee.

Inclusion criteria were patients with lower urinary tract symptoms due to BPH and indications for the surgical treatment. Men with a history of prostate cancer, urethral stricture, bladder neck sclerosis, and neurogenic bladder were excluded from analysis. Anticoagulant therapy for patients in the group was not discontinued before surgery.

The perioperative outcomes included operating time, laser working time, applied energy, length of catheterization and hospital stay. Serum sodium and hemoglobin level were also evaluated before and after the operation. The prostate size was measured by transrectal ultrasonography (TRUS). All patients were reevaluated at 1, 3, 6, and 12 months postoperatively and adverse events were also assessed. Functional evaluation such as International Prostate Symptom Score (IPSS), maximum flow rate (Qmax), post-void residual urine (PVR), prostate-specific antigen (PSA) level, and prostate volume were measured at baseline and each point.

Statistical analysis was performed using the SPSS 11.5 software package. Data was expressed as mean ± standard deviation. Ordinal data was analyzed using the Student’s t test when comparisons were made and p value of less than 0.05 was considered to be statistically significant.

## Surgical technique

**Fig. 1:**
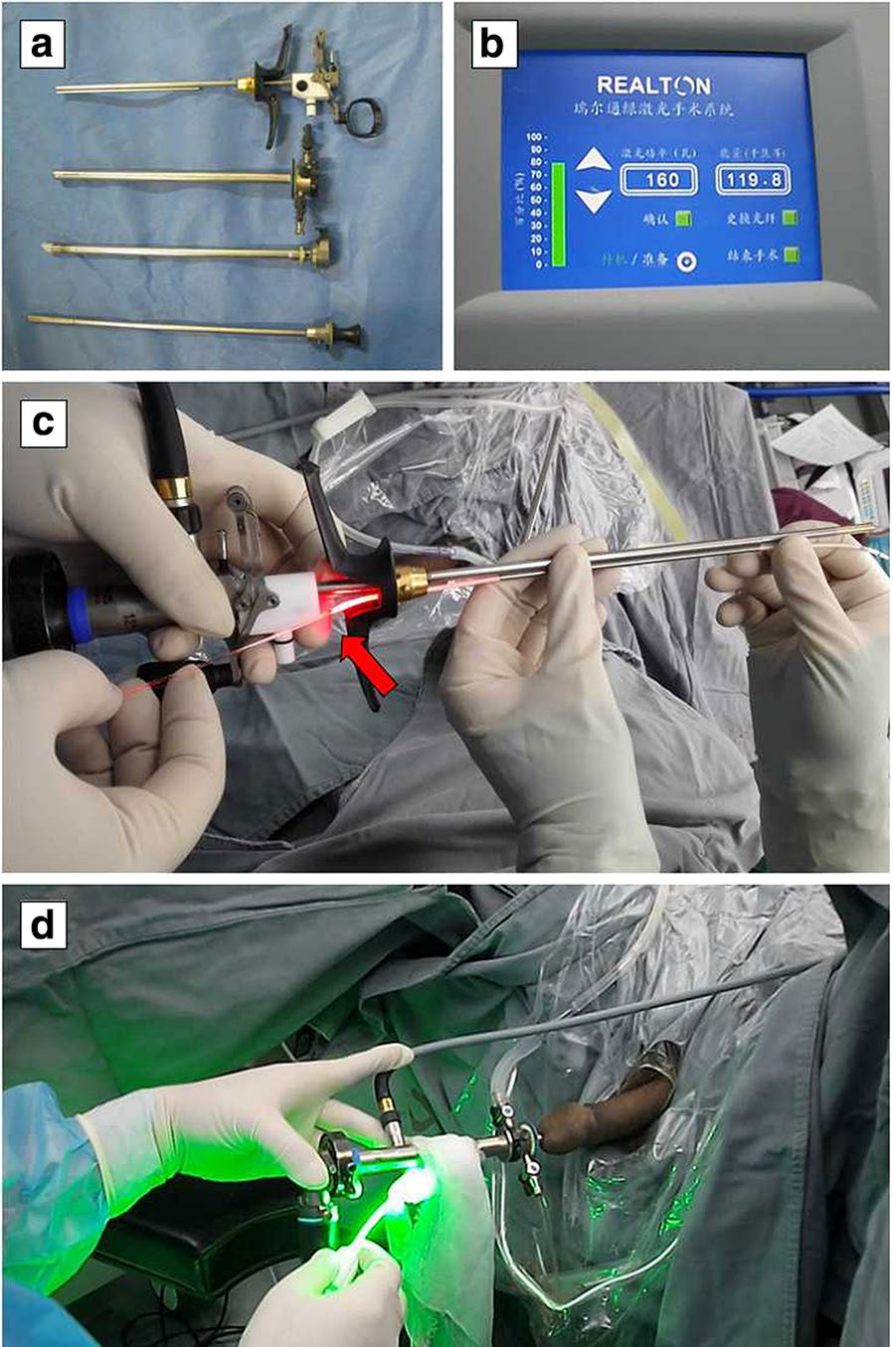
**a** The 26-Fr Sheath of continuous flow plasma kinetic resectoscope for PRSVP. **b** The user interface of 160-W LBO laser generator. **c** Using the ureteral stent for directing the laser fiber inside the channel. **d** Hand manipulation for the PRSVP

Related surgical devices for preoperative preparation were listed in Table 1. Before the start of surgery, we need to use the ureteral stent for directing the laser fiber inside the ring channel of plasma resectoscope in order not to damage the channel (Fig. 1).

**Table 1:** Surgical equipments in Shandong Provincial Hospital

160 W LBO laser generator (Beijing Realton Laser Technology Co. Ltd. China)
Straight Beam LBO Laser fiber (Beijing Realton Laser Technology Co. Ltd. China)
26-Fr Sheath of continuous flow plasma kinetic resectoscope (Olympus Corp. Japan)
12° Telescope, Ellik evacuator device (Olympus Corp. Japan)
Endoscopic camera and Video tower (Olympus Corp. Japan)
Ureteral stent (Guide the laser fiber into the ring channel of resectoscope)
Extract forceps (Clamp the specimen of prostate tissue)

*LBO* lithium triborate

- *Step 1* Start stripping-vaporization from both sides of the verumontanum

**Fig. 2:**
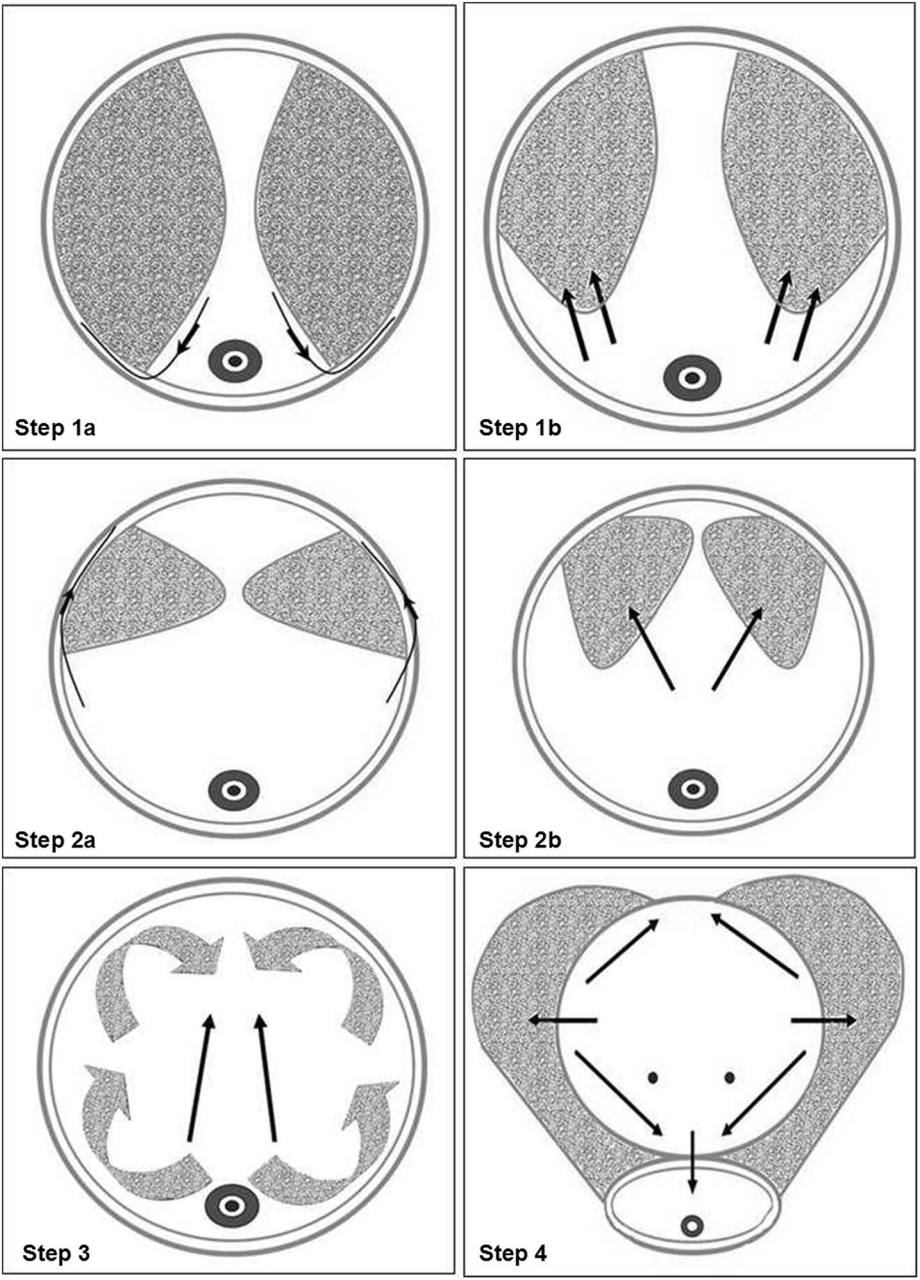
Procedures of the PRSVP. **Step 1a** Stripping the prostate tissue to the prostatic capsula from the verumontanumon to both sides between the 9- and 3-o’clock positions. **Step 1b** Vaporizing the stripped prostate tissue between the 9- and 3-o’clock positions. **Step 2a** Stripping the prostate tissue of the 12 o’clock direction to the prostatic capsula. **Step 2b** Vaporizing the stripped prostate tissue of the 12 o’clock direction. **Step 3** Inward and holistically stripping-vaporization of the whole prostate. **Step 4** Stripping-vaporization around the bladder neck for a circle

The first thing is to observe the situation of prostatic hyperplasia and bilateral ureteral orifice. If a lobe of the prostate protruding into the bladder obviously, we can start the surgery no matter we could watch the orifice. We begin to strip and vaporize the prostate tissue with 160-W power from the verumontanum to both sides between the 9- and 3-o’clock positions. For large prostates, we need to vaporize some prostatic tissue around the verumontanum firstly to make a space for stripped prostate tissue in the verumontanum position (Fig. 2: Step 1a and Step 1b).

- *Step 2* Stripping-vaporization of the 12 o’clock direction

As both lateral lobes of the prostate are gradually vaporized, the previous urethra turn to be wider than before. Meanwhile the gland of 12-o’clock position gradually prominent obviously to the below urethra. We go on vaporizing the prostatic tissue of 12-o’clock position along the prostatic capsule which was in the same stripped plane of 9- and 3-o’clock positions toward the bladder neck. When vaporizing the prostatic tissue of 12-o’clock position, we need to rotate sheath in order to achieve direct vaporization with almost vertical angle (Fig. 2: Step 2a and Step 2b).

- *Step 3* Inward and holistically stripping-vaporization of the whole prostate

Both lateral lobes of the prostate are retrograde stripped to the prostatic capsule and then vaporized from the verumontanum to bladder neck. The stripped prostatic tissue will be vaporized with less bleeding for less blood supply. During the procedures, the fiber could face the vaporized tissue by contact-type without of angle (Fig. 2: Step 3).

- *Step 4* Stripping-vaporization around the bladder neck for a circle

Stripping-vaporization around the bladder neck should be taken to prevent injury of bilateral ureteral orifice. Gradually we firstly make an incision at 6-, 3- and 9-o’clock position so that the triangular area of bladder and the prostatic department of urethra are in the same horizontal plane. Then we can perform stripping and vaporization to whole bladder neck ring along the incision plane of 6-, 3- and 9-o’clock position. For the prostate which are smaller but obstructive symptoms are more severe, we will make incision at 5- and 7-o’clock position at the same time (Fig. 2: Step 4).

- *Step 5* Completion of stripping-vaporization and take pathological specimens

**Fig. 3:**
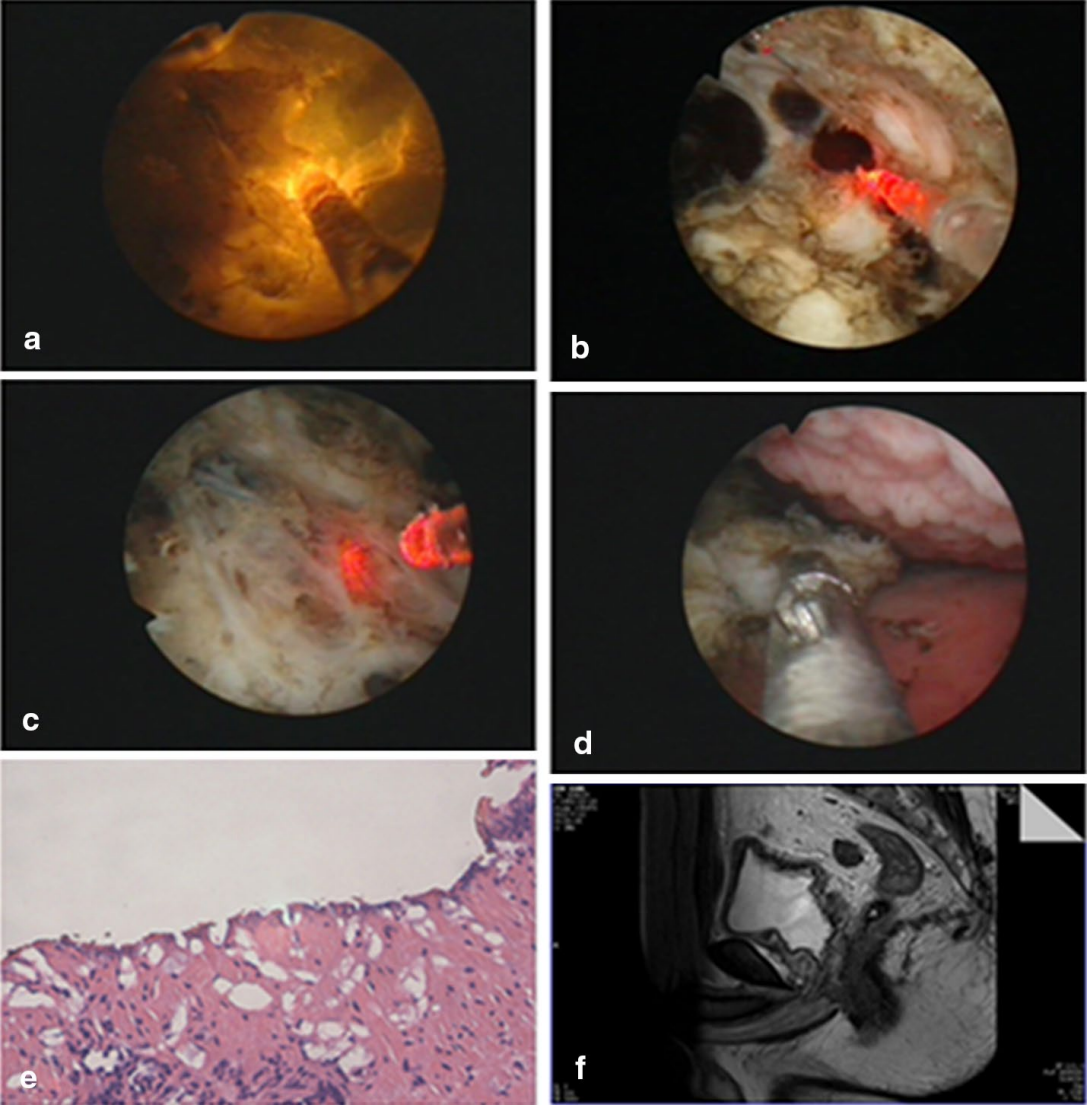
**a** Showing the straight beam working type of green laser. **b** Stripping the prostate tissue. **c** Showing the prostatic capsula. **d** Taking pathology specimens during the operation. **e** Pathological picture shows the prostate hyperplasia (HE staining ×10). **f** Prostate MRI image after surgery

Then we will dressing the entire urethra so that make a spacious and smooth channel. And now we can select a location for taking pathology specimens. Specimens or blocks of prostate can be taken out with Ellik evacuator, or forceps for smaller tissue piece. Finally a catheter will be indwelt with balloon water of 40–60 ml. No need of postoperative continuous bladder irrigation and catheter traction (Fig. 3).

## Results

All the 180 patients were identified forming the cohort for this study. Table 2 provides a summary of baseline characteristics for the patients. The median age was 70.2 years (range 60.9–79.4). Of all the patients, 56 had a history of urinary retention and 44 were taking antiplatelet and anticoagulant medication.

**Table 2:** Baseline patient characteristics

Parameter	Value
Age (years)	70.2 ± 9.3 (60.9–79.4)
TRUS prostate volume (ml)	66.5 ± 35.3 (32.3–100.8)
PSA (ng/ml)	4.8 ± 2.2 (2.6–6.9)
Qmax (ml/s)	6.5 ± 5.4 (4.3–10.6)
IPSS	24.5 ± 5.2 (19.3–29.6)
PVR (ml)	167.5 ± 158.9 (132.6–203.3)
Patients [n (%)]	
Anticoagulant medication	44 (24.4 %)
Urinary retention requiring catheterization	56 (31.1 %)
Prostate biopsy before surgery	50 (27.8 %)

Results are reported as mean ± standard deviation (range)

*TRUS* transrectal ultrasound, *PSA* prostate-specific antigen, *Qmax* maximum flow rate, *IPSS* International Prostate Symptom Score, *PVR* post-void residual urine

Perioperative outcomes are reported in Table 3. The mean laser working time was 48.3 ± 14.4 min and the mean energy applied was 315.2 ± 122.6 kJ. None of the patients required postoperative irrigation. The mean catheterization time was 1.5 ± 0.8 days and the mean postoperative hospital stay time was 2.6 ± 1.0 days. Two patients (1.1 %) were diagnosed with prostate cancer by postoperative histopathology examination. For operative time, laser working time and energy usage, there were four values missing due to incomplete data recording. For post-operative length of catheterization and post-operative duration of hospital stay, there were six values missing for the same reason.

**Table 3:** Perioperative outcomes

	Mean ± SD	Range
Operative time (min)	62.2 ± 16.5	45.8–77.9
Laser working time (min)	48.3 ± 14.4	35.2–62.2
Applied energy (kJ)	315.2 ± 122.6	193.6–433.1
Serum sodium (mmol/l)		
Preoperative	136.5 ± 3.2	133.4–139.6
Postoperative	134.5 ± 2.8	131.9–137.2
p value	0.548	
Hemoglobin (g/dl)		
Preoperative	13.5 ± 1.7	11.8–15.0
Postoperative	13.2 ± 1.5	11.7–14.6
p value	0.534	
Catheterization time (day)	1.5 ± 0.8	0.7–2.2
Postoperative hospital stay time (day)	2.6 ± 1.0	1.6–3.5

Results are reported as mean ± standard deviation (range)

Functional outcomes in terms of IPSS, Qmax, PVR, reductions in PSA and prostate size at baseline and 1, 3, 6, 12 months post-operation, respectively, are shown in Table 4. The data clearly demonstrate that IPSS, Qmax and PVR were dramatically improved at 1, 3, 6, and 12 months after PRSVP (p < 0.01). Compared with preoperative values, mean prostate volume and serum PSA fell by 63.0 and 60.4 %, respectively, at 1 month after PRSVP.

**Table 4:** Preoperative and postoperative functional parameters

Parameter	Baseline	1 Month	3 Months	6 Months	12 Months
IPSS	24.5 ± 5.2	7.5 ± 2.8*	6.2 ± 2.6*	7.0 ± 2.3*	6.5 ± 2.1*
Qmax (ml/s)	6.5 ± 5.4	19.5 ± 8.2*	20.6 ± 7.1*	22.4 ± 5.4*	21.3 ± 3.8*
PVR (ml)	167.5 ± 158.9	30.2 ± 28.5*	26.2 ± 17.6*	20.5 ± 14.3*	16.8 ± 11.0*
PSA (ng/ml)	4.8 ± 2.2	1.9 ± 1.7*	1.4 ± 1.5*	1.5 ± 1.2*	1.7 ± 1.6*
PV (ml)	66.5 ± 35.3	24.6 ± 10.5*	22.9 ± 8.1*	23.7 ± 9.2*	25.4 ± 9.5*

Results are reported as mean ± standard deviation

*IPSS* International Prostate Symptom Score, *Qmax* maximum flow rate, *PVR* post-void residual urine, *PSA* prostate-specific antigen, *PV* prostate volume

* Highly significant difference compared to baseline, p < 0.005

Adverse events are shown in Table 5. Complications were categorized as intraoperative, early (<30 days), or late (>30 days) postoperative complications. Complications were few. None of the patients required blood transfusion. Capsular perforation was found in four patients during PRSVP for both the patients had small prostate volume less than 30 ml, and two patients experienced bleeding from prostatic venous sinus. Neither bladder wall nor ureteral orifice was injured in the patients. Urinary tract infections with signs of bacteremia were seen in nine patients within 1 month of PRSVP. Four patients experienced bladder neck contracture undergoing bladder neck incision at 6 months after operation and has subsequently done well. No dysuria and secondary haemorrhage was observed during the follow-up period. Urethral stricture was diagnosed in four patients, who were treated by dilatation. None of the patients required reoperation because of regrowth of prostate.

**Table 5:** Intraoperative, early, and late postoperative complications classified by Clavien–Dindo grade

Complication	Grade	Number of cases (%)
*Intraoperative complications*		
Capsule perforation	3b	4 (2.2 %)
Bleeding from prostatic venous sinus	1	2 (1.1 %)
Ureteric Orifice injury	2	2 (1.1 %)
Blood transfusion	1	0 (0.0 %)
*Early, and late postoperative complications*		
Transient urinary retention	1	6 (3.3 %)
Urinary tract infection	2	9 (5.0 %)
Bladder neck contracture	3a	4 (2.2 %)
Secondary haemorrhage	1	2 (1.1 %)
Urethral stricture	2	4 (2.2 %)
Reoperation	3a	0 (0.0 %)

## Discussion

Currently, TURP is still the gold standard in the surgical treatment of BOO caused by BPH. The rapid development of laser technology makes PVP be widely recognized (Bach et al. 2012). Three clinical randomized trials have demonstrated that PVP at least have equally efficacy and safety compared with TURP, especially for the elderly and high-risk patients with oral anticoagulation and bleeding tendency (Chen et al. 2012; James et al. 2015; Teng et al. 2013).

Until now, there has been little experience reported on the performance of the straight beam green laser vaporesection in the treatment of BPH (Gong et al. 2014). The GOLIATH study carried out by James A. Thomas et al. show that green light laser vaporization with 180 W XPS system is safe and effective as TURP for BPO, according to 2-year follow-up data (James et al. 2015). Even so, the confirmed shortcomings of PVP include slower vaporization speed, uncontrollable bleeding during operation, poor visibility and recurrence of residual gland postoperative (Spaliviero et al. 2008). In terms of the surgical instruments and operating procedures, some conventional methods, such as vaporizing the prostate lobe by lobe and vaporization-resection of prostate tissue, have been widely reported (Netsch and Bach 2015). Misrai et al. have raised up the “En Bloc” enucleation of the prostate using a surgical 532-nm laser (GreenLEP) technique, which need a morcellator for morcellation of prostate tissue (Misrai et al. 2015). However, the ideal surgical approach has not yet appeared.

On the basis of operational experience of PVP over 10 years, we not only improve the assembly of operation instrument scientifically by making full use of the existing equipment available, but also put forward our own surgical technology of retrograde stripping-vaporization using straight beam green laser. We analyzed the statistical data and achieved good results.

Following aspects are the main innovations and research findings: (1) Switching to plasma kinetic resectoscope in place of traditional sheath in the PRSVP to improve the effect of flushing water. It will increase water flow of bladder irrigation during operation. In addition, we also developed the method of putting the LBO laser fiber into the resectoscope sheath. (2) Improvement of vaporization method with straight beam LBO laser fiber. During the operation, we perform stripping-vaporizing the prostatic tissue by the way of stripping simultaneously vaporization. We use contact type vaporization during the operation. When there is a bleeder, using the laser fiber tip to vaporize the bleeder directly and lowing down the power to about 30–60 W can stop the bleeding. (3) Traditional PVP operation cannot acquire pathological specimen (Malek et al. 2005). The improved PRSVP can obtain pathological specimens from the surgical capsule of prostate to avoid the omission of prostate cancer.

For large prostates, especially in those larger than 100 g, there is no need to strip the tissue directly reaching the surgical capsule at the beginning of the operation (Kim et al. 2015). Firstly, we usually vaporize the tissue around the verumontanum to achieve a space for the next further stripping to capsule.

In patients with small prostates, we can directly strip the prostate to the surgical capsule. Actually we have been vaporizing a wall of prostate tissue and will create a more spacious urethra during the operation. So there is obvious superiority by stripping-vaporizing than single enucleation which will narrow operation space (Raison and Challacombe 2015).

In this study, the perioperative data show that there is small fiber power loss, high vaporization efficiency and short operative time in the PRSVP. In comparing our operative time to the previous study by Gong YG and James A, we have experienced slightly longer operating time, perhaps because the prostate volume was larger in our study (Gong et al. 2014; James et al. 2015). The follow-up results strongly suggest that the effect of PRSVP is lasting. IPSS score decrease continuously; Qmax and PVR improve continuously as well. The prostate volume has no recurrence of hyperplasia even reoperation required over 1 year of follow-up. The data show that the direct clinical effect of PRSVP can be stable and sustainable, even further more improvement during the follow-up.

In terms of surgical complications, intraoperative blood loss is less in patients without blood transfusions, especially for the patients with oral anticoagulant therapy. The surgery has so exact security in blood loss that the catheter time and postoperative time of hospital stay are all significantly reduced. The patients with oral anticoagulation do not need to discontinue the anticoagulant drug, and there is no significant risk of secondary hemorrhage.

The capsule perforation usually require surgical, endoscopic, or radiologic intervention, so it is Clavien 3b. There were four patients (2.2 %) who had capsule perforation in our study, which is less common with those described following TURP (compare with 4 % incidence with TURP) and consistent with PVP using the 180- or 120-W LBO lasers (Mandal et al. 2013; Rieken et al. 2010; Campbell et al. 2013). We performed a conservative treatment by the pulling of the catheter without re-operated.

Bladder cramps is one of common complications after surgery. There are many factors that can cause bladder cramps, rinses inappropriate temperature is one of the main reasons. In this study, all the patients did not use bladder irrigation after the operation, so the bladder spasm is rare.

The procedure does not increase the risk of urethral stricture and bladder neck contracture. There are four cases of bladder neck contracture. All of the four patients have a small volume of prostate. This complication associated with minor volume of prostate and incision of the bladder neck tissue. In this study, the incidence of bladder neck sclerosis and urethral stricture are comparable to those previously reported in studies on PVP and of low incidence (Rieken et al. 2010). Urethral stricture may due to a plasma resectoscope sheath (26F) is used, which has larger diameter than traditional PVP sheath. The bladder neck sclerosis and urethral stricture were all mild and short like thin membrane and dialatation management was enough without a bladder neck incision or urethrotomy.

The rate of minor complications is similar with previous literature reports (Gong et al. 2014; Chen et al. 2012). The surgical approach does not increase the occurrence of injury to the external urethral sphincter which could result in real incontinence. There is no case of damage to the bilateral ureteral orifices.

## Limitation of the study

The main limitations were a short follow-up period and the absence of a comparison arm. However, considering that we focus on a new surgical approach and provide an early experience, 12 months follow-up seems to be acceptable. Besides, the effect of PRSVP on sexual function remain to be explored. Further data from either large prospective trials or long-term follow-up is required.

## Conclusion

Based on our researches and practices, the 160-W straight beam LBO laser PRSVP is effective as a treatment option for symptomatic BPH. This technology could offer the possibility of more rapid tissue vaporization. Further substantiation of these results by data from either large prospective trials or long-term follow-up is required.
